# *Vibrio vulnificus* induces mTOR activation and inflammatory responses in macrophages

**DOI:** 10.1371/journal.pone.0181454

**Published:** 2017-07-18

**Authors:** Dan-Li Xie, Meng-Meng Zheng, Yi Zheng, Hui Gao, Jie Zhang, Ting Zhang, Jian-Chun Guo, X. Frank Yang, Xiao-Ping Zhong, Yong-Liang Lou

**Affiliations:** 1 Department of Microbiology and Immunology, School of Laboratory Medicine, Wenzhou Medical University, Wenzhou, Zhejiang, China; 2 China Ministry of Education Key Lab of Laboratory Medicine, Wenzhou, Zhejiang, China; 3 Department of Clinical Laboratory Medicine, Sichuan Provincial People’s Hospital, Chengdu, Sichuan, China; 4 Department of Laboratory Medicine, Jinshan Hospital of Fudan University, Jinshan, Shanghai, China; 5 Department of Microbiology and Immunology, Indiana University School of Medicine, Indianapolis, Indiana, United States of America; 6 Department of Pediatrics, Division of Allergy and Immunology, Duke University Medical Center, Durham, NC, United States of America; Ohio State University, UNITED STATES

## Abstract

*Vibrio vulnificus* (*V*. *vulnificus*), a Gram-negative marine bacterium, can cause life-threatening primary septicemia, especially in patients with liver diseases. How *V*. *vulnificus* affects the liver and how it acts on macrophages are not well understood. In this report, we demonstrated that *V*. *vulnificus* infection causes a strong inflammatory response, marked expansion of liver-resident macrophages, and liver damage in mice. We demonstrated further that *V*. *vulnificus* activates mTOR in macrophages and inhibition of mTOR differentially regulates *V*. *vulnificus* induced inflammatory responses, suggesting the possibility of targeting mTOR as a strategy to modulate *V*. *vulnificus* induced inflammatory responses.

## Introduction

*Vibrio vulnificus* (*V*. *vulnificus*) is a halophilic, Gram-negative and life-threatening marine bacterium [1, 2]. *V*. *vulnificus* infection can cause severe primary septicemia in patients with liver diseases such as viral hepatitis, advanced cirrhosis, and chronic alcoholic liver disease [3]. Despite previous studies have significantly advanced our understanding of the pathogenesis of *V*. *vulnificus* infection, mechanisms that regulate *V*. *vulnificus*-mediated responses remain unclear.

Upon infection, innate immune cells sense the pathogen and initiate cascades of events that result in the production of cytokines such as IL-1β, IL-6, and TNFα, which recruit other immune cells such as macrophages, neutrophils, and dendritic cells (DCs) to defend against pathogen invasion [4, 5]. Toll-like receptors (TLRs) or Nod-like receptors (NLRs) recognition of bacterial components activates NF-κB signaling, which is important for transcription of many cytokines [6]. In addition, activation of inflammasomes, marked by caspase-1 activation and IL-1β secretion [7–10], is an important aspect of innate response to danger signals during bacterial infection. Mammalian target of rapamycin (mTOR) is a serine/threonine kinase that regulates both adaptive and innate immune cell development, metabolism, and function [11–16]. mTOR forms two distinct signaling complexes, mTORC1 and mTORC2. Activated mTORC1 phosphorylates multiple downstream molecules such as S6K1 and 4E-BP1. mTORC2 phosphorylates Akt at serine 473 to regulate cell survival and nutrient uptake [17–19]. mTORC1, but not mTORC2, is sensitive to acute rapamycin treatment. Recent studies have found that mTOR and its tight regulation play important roles in innate immune responses [11, 15, 16, 20–22]. Many extracellular signals induce mTORC1 and mTORC2 activation in innate immune cells, including TLR, cytokines, and growth factors [15, 20, 23]. mTORC1controls macrophage polarization [12, 21], is important for alveolar macrophage self-renewal [24], and regulates dendritic cell metabolism, maturation, and function [25–27]. Whether *V*. *vulnificus* can induce mTOR activation and how mTOR may regulate innate immune responses to this pathogen in macrophages is unknown.

In this report, we demonstrated that *V*. *vulnificus* could directly inflict liver damage and induce strong innate immune responses and Kupffer cell expansion. Moreover, *V*. *vulnificus* can activate mTOR in macrophages and inhibition of mTOR differentially regulates *V*. *vulnificus* induced inflammatory responses.

## Results

### Acute infection by *V*. *vulnificus* CGMCC1.1758 strain induced liver inflammation in C57BL/6J mice

**Fig 1:**
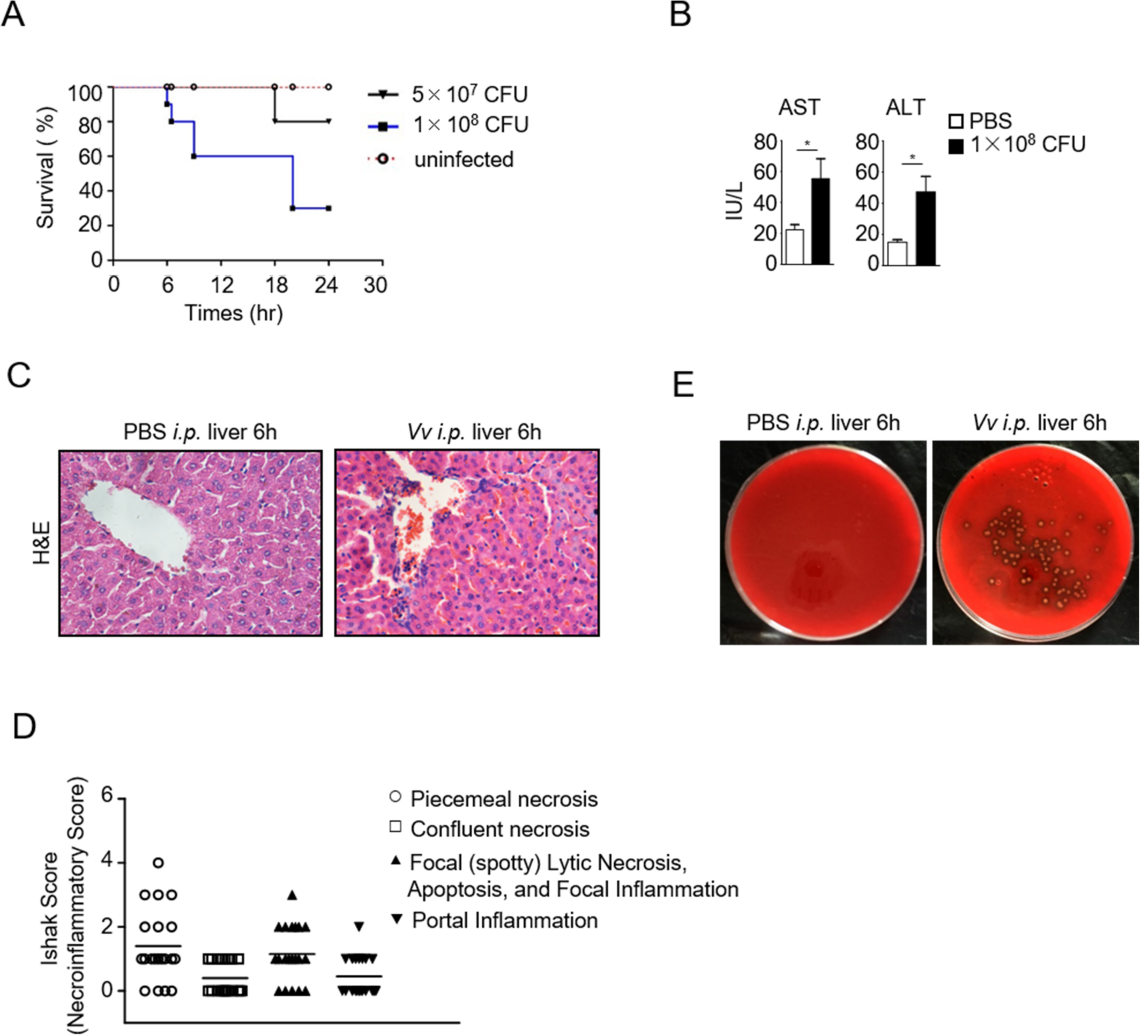
Induction of liver inflammation by *V*. *vulnificus* CGMCC1.1758 strain acute infection. (A) Survival curves for 5–6-week-old C57BL/6J mice by i.p. injection of 5 × 10^7^ CFU or 1 × 10^8^ CFU of *V*. *vulnificus* (CGMCC1.1758 strain). *P*<0.01 between uninfected group and 1 × 10^8^ CFU group assessed with the log-rank survival analysis (n = 10 in each group). (B) Serum AST and ALT concentrations in uninfected and 1 × 10^8^ CFU *V*. *vulnificus*-infected mice 6 hours after infection. The values are the means ± SEM of at least triplicate samples (n = 4). *, *P*<0.05 determined by Student’s *t*-test. (C) H&E stain of liver thin sections from control and 1 × 10^8^ CFU of *V*. *vulnificus* group 6 hours after injection. (D) Ishak score of liver inflammation in *V*. *vulnificus*-infected mice. The scores of all control mice were zero, and were not shown. (E) Detection of *V*. *vulnificus* after overnight culture of liver homogenates in BHI rabbit blood plates. We prepared liver homogenates 6 hours after 1 × 10^8^ CFU *V*. *vulnificus* infection. Data shown are representative of at least three experiments.

Patients with liver diseases are susceptible to severe *V*. *vulnificus* infection. One of the liver’s key functions is to clear blood-borne pathogens and to prevent bacteria from spreading to other organs to cause sepsis [28]. Several strains of *V*. *vulnificus* have been found to cause systemic infection in mice [29–31]. However, whether the *V*. *vulnificus* 1.1758 strain can cause liver damage has been unclear. To examine whether *V*. *vulnificus* is capable of causing liver damage, we intraperitoneally (i.p.) injected *V*. *vulnificus* CGMCC1.1758 into wild-type C57BL/6J mice. Mice injected with 1×10^8^ CFU *V*. *vulnificus* showed higher mortality than those injected with 5×10^7^ CFU (Fig 1A). *V*. *vulnificus* infection resulted in liver damage, indicated by elevated serum alanine aminotransferase (ALT) and aspartate aminotransferase (AST) levels (Fig 1B). H&E staining showed piecemeal necrosis, focal inflammation, and portal inflammation in the liver (Fig 1C and 1D). Additionally, we detected *V*. *vulnificus* in liver homogenates (Fig 1E). These observations indicated that *V*. *vulnificus* CGMCC1.1758 is capable of inducing liver inflammatory damage in wild-type C57BL/6J mice.

### *V*. *vulnificus* acute infection-induced Kupffer cell and neutrophil accumulation in the liver

**Fig 2:**
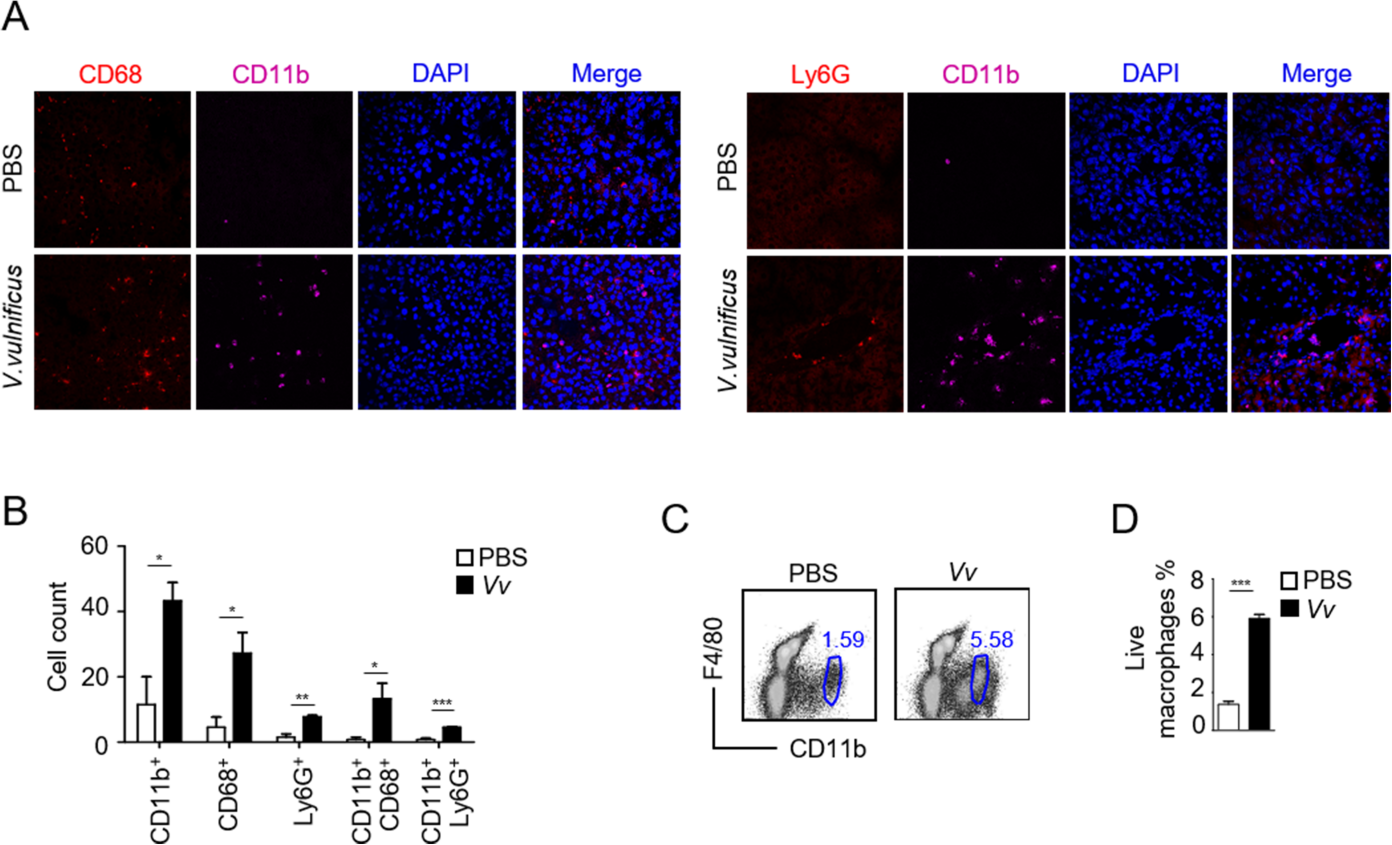
Accumulation of Kupffer cells and neutrophils in the liver after acute *V*. *vulnificus* infection. We injected C57BL/6J mice with PBS or 1 × 10^8^ CFU of *V*. *vulnificus*. Six hours later, we harvested livers for analysis. (A) Confocal microscopy detection of Kupffer cells (CD68^+^CD11b^+^) and neutrophils (Ly6G^+^CD11b^+^) in frozen liver sections (magnification: 400×). (B) Bar figures show mean ± SEM of numbers of indicated cells in 4 fields from *V*. *vulnificus*-infected mice (n = 4). Cell count was analyzed by Nikon NIS Elements software with General Analysis Plug. (C) Representative dot plots showing F4/80 and CD11b staining in liver mononuclear cell preparations. (D) Bar graphs show the frequency of CD11b^int^F4/80^+^ liver macrophages subsets. The values are the means ± SEM (n = 6). Data shown are representative of at least three experiments. ***, *P*<0.001 as determined by Student’s *t-*test.

To determine the infiltrating cell types in the liver after *V*. *vulnificus* infection, we performed immunofluorescence analysis of frozen liver sections of *V*. *vulnificus*-infected and control mice. As Fig 2A and 2B shows, both CD68^+^CD11b^+^ Kupffer cells and CD11b^+^Ly6G^+^ neutrophils accumulated in the livers of infected mice. To further confirm Kupffer cell accumulation, we performed FACS analysis of liver mononuclear cells. As shown in Fig 2C and 2D, CD11b^+^F4/80^int^ Kupffer cells were considerably increased in *V*. *vulnificus* infected mice. Thus, acute *V*. *vulnificus* infection caused Kupffer cell accumulation in the liver.

### *V*. *vulnificus* acute infection-induced Kupffer cell proliferation and proinflammatory responses in vivo

**Fig 3:**
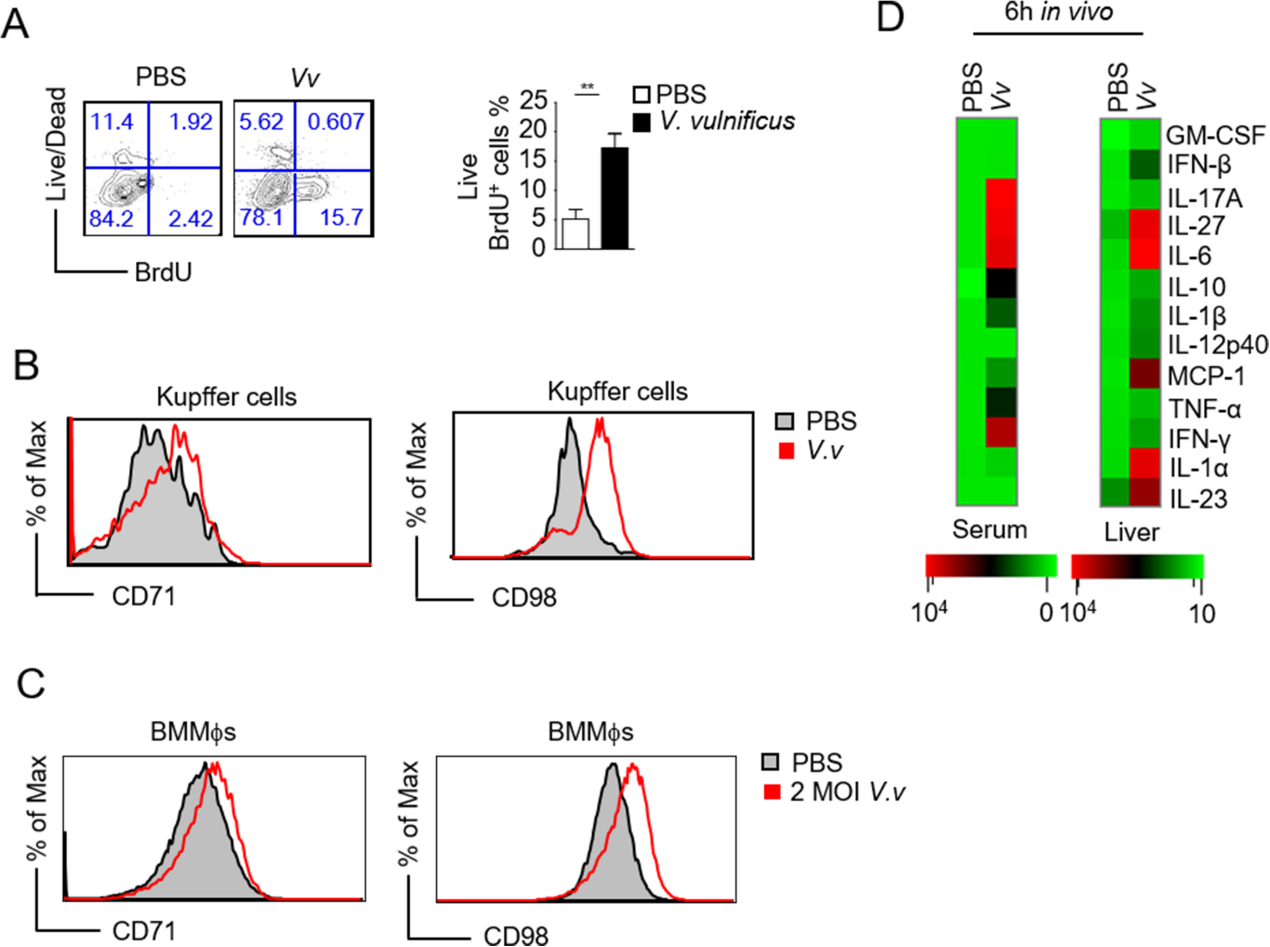
Increased Kupffer cell proliferation and proinflammatory cytokines production after acute *V*. *vulnificus* infection. We injected BrdU into C57BL/6J mice and then with PBS or 1 × 10^8^ CFU of *V*. *vulnificus*. Six hours later, we harvested livers for analysis. (A) The contour plot and bar graph show the frequency of BrdU-incorporated CD11b^int^F4/80^+^ liver macrophages (n = 3). (B) CD71 and CD98 expression on CD11b^int^F4/80^+^ macrophages. (C) CD71 and CD98 expression in live CD11b^+^F4/80^+^ BMMϕs after 6 hours in vitro *V*. *vulnificus* infection. (D) Cytokine expression profile in serum and liver homogenates from 1 × 10^8^ CFU *V*. *vulnificus*-infected mice and control mice 6 hours after infection measured by the multiplex flow assay (Biolegend). The values are the means ± SEM (n = 3). Data shown are representative of at least three experiments. **, *P*<0.01 as determined by Student’s *t-*test.

To determine whether Kupffer cell accumulation in the liver after *V*. *vulnificus* infection was associated with increased proliferation, we injected BrdU into mice immediately before *V*. *vulnificus* infection. Six hours after injection, Kupffer cells from *V*. *vulnificus*-infected livers incorporated more BrdU than those in uninfected mice, suggesting that these cells proliferated in infected mice (Fig 3A). Kupffer cells from *V*. *vulnificus*-infected mice upregulated CD71 (transferrin receptor protein 1, required for iron delivery from transferrin to cells) and CD98 (an amino acid transporter) (Fig 3B). In vitro, *V*. *vulnificus* infection of BMMϕs also upregulated CD71 and CD98 (Fig 3C), suggesting that *V*. *vulnificus* directly acted on macrophages to induce expression of these molecules. Elevated expression of CD71 and CD98 might ensure sufficient nutrients for macrophage metabolism, proliferation, and function. Together, these observations suggest that acute *V*. *vulnificus* infection caused Kupffer cells to accumulate in the liver by inducing their proliferation.

To determine if *V*. *vulnificus* induced inflammatory responses, we measured cytokines concentrations in the serum and liver homogenates after *V*. *vulnificus* infection using a multiplex flow assay. In *V*. *vulnificus*-infected mice, multiple cytokines, such as IL-27, IL-6, IL-10, and MCP-1, obviously increased in both blood and liver (Fig 3D). Several other cytokines, such as IFN-β, IL-23, IL-12 p40, and IL-1α, increased only in the liver, while TNF-αwas increased in serum, but not in the liver. The differences between liver and blood could result from differences in bacterial burdens and in local immune cell compositions in these organs/tissues. Thus, *V*. *vulnificus* infection triggered strong innate immune responses in vivo.

### Activation of NFκB and mTOR in macrophages by *V*. *vulnificus*

**Fig 4:**
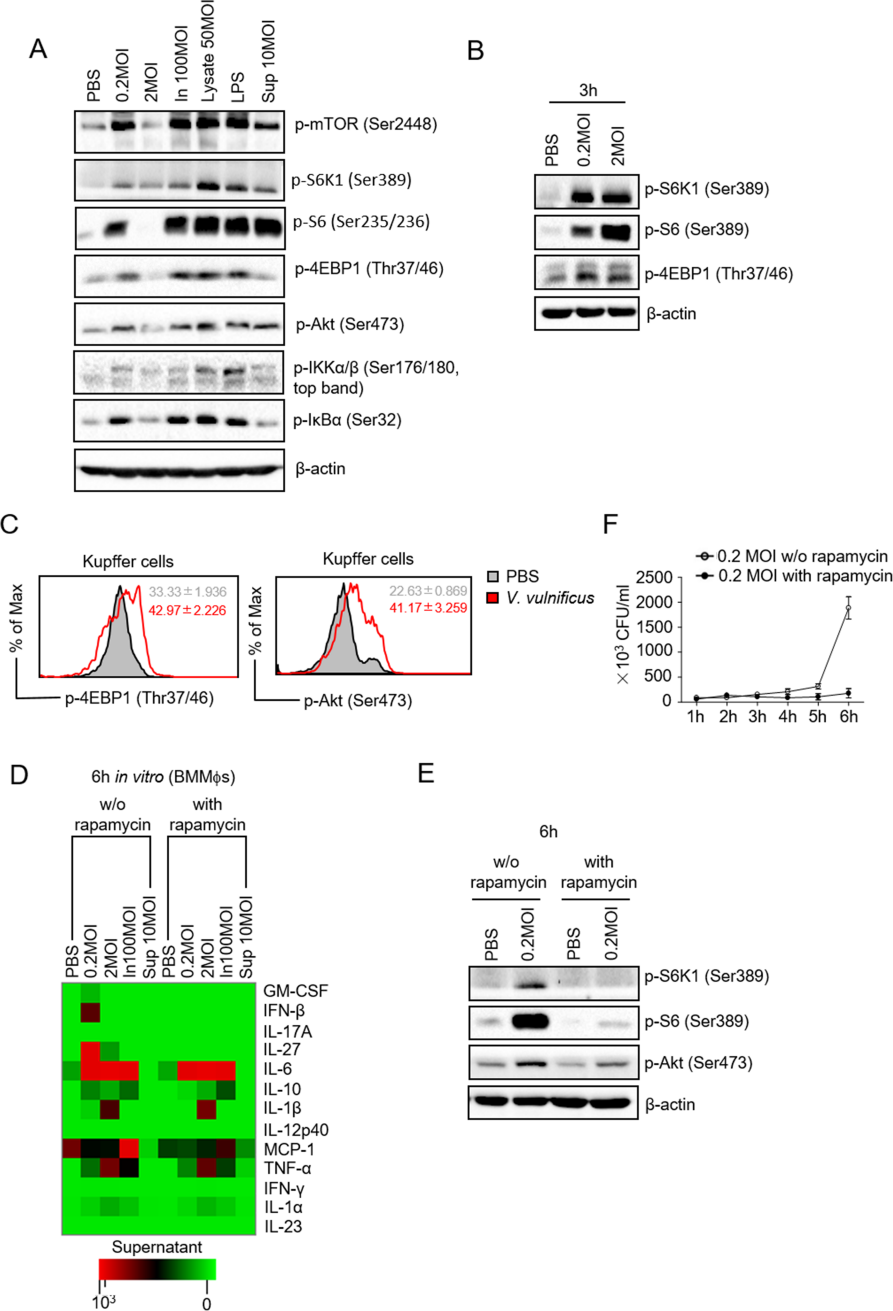
Effects of *V*. *vulnificus* inducted mTOR activation on innate immune responses in macrophages. (A) Detection of protein phosphorylation in BMMϕ treated with live or heat-inactivated *V*. *vulnificus*, its lysates, or culture supernatant for 6 hours. (B) Detection of protein phosphorylation in BMMϕ treated with 0.2 or 2.0 MOI *V*. *vulnificus* for 3h. (C) Detection of 4E-BP1 (Thr37/46) and Akt (Ser473) phosphorylation by intracellular staining and FACS analysis in CD11b^int^F4/80^+^cells isolated from liver of after infection of mice with 1 × 10^8^ CFU *V*. *vulnificus* (n = 3). Means ± SEM in the plots show MFI of 4E-BP1 (Thr37/46) and Akt (Ser473) phosphorylation. (D) Cytokine profile in the supernatants of BMMϕ after in vitro *V*. *vulnificus* infection for six hours in the presence or absence of 100 nM rapamycin. (E) Detection of protein phosphorylation in BMMϕ treated by 0.2 MOI *V*. *vulnificus* for 6h in the presence or absence of 100nM rapamycin. (F) Growth-curve of *V*. *vulnificus* in the supernatant of BMMϕs infected with 0.2 MOI *V*. *vulnificus*in the presence or absence of 100nM. Data shown represent three experiments.

Transcription of many cytokines depends on NFκB after stimulating pathogen pattern recognition receptors [5, 32]. Bone marrow derived macrophages (BMMϕs) infected with live 0.2 MOI *V*. *vulnificus or* treated with heat inactivated *V*. *vulnificus* (In) or *V*. *vulnificus* lysates for 6 hours contained increased IKKα/β and IκBα phosphorylation (Fig 4A), suggesting that *V*. *vulnificus* was capable of triggering IKK-NFκB activation. Low dose (0.2 MOI) *V*. *vulnificus* treatment also induced phosphorylation of mTOR, S6K1, S6, 4EBP1 and Akt. However, 2 MOI of *V*. *vulnificu*s-treated cells did not induce phosphorylation of these proteins (Fig 4A), which was likely caused by negative feedback mechanisms triggered by over-stimulation or by death of BMMϕs after 6 hours of infection. Indeed, after 3 hours treatment, 2 MOI *V*. *vulnificus* induced stronger S6K1 and S6 phosphorylation than 0.2 MOI *V*. *vulnificus* (Fig 4B). Moreover, Kupffer cells from *V*. *vulnificus*-infected mice also contained increased 4E-BP1 and Akt S473 phosphorylation (Fig 4C). These observations suggested that *V*. *vulnificus* induced both mTORC1 and mTORC2 activation in macrophages in vitro and in vivo.

To examine if *V*. *vulnificus* could directly induce innate immune responses in macrophages, we treated BMMϕs with live *V*. *vulnificus* or heat inactivated *V*. *vulnificus*, or *V*. *vulnificus* culture supernatant (Sup) in vitro for 6 hours. As Fig 4D shows, both live and heat-inactivated *V*. *vulnificus* induced IL-6, IL-10, TNF-α, and IL-1α production in these cells. In addition, live but not inactivated *V*. *vulnificus* induced GM-CSF, IFNβ, IL-27, and IL-1β production. Interestingly, treatment of BMMϕs with rapamycin during *V*. *vulnificus* stimulation resulted in decreased expression of GM-CSF, IFNβ, IL-27, and MCP-1 without obviously affecting the production of other cytokines. In rapamycin treated BMMϕs, *V*. *vulnificus*-induced S6K1 and S6 phosphorylation was abolished and Akt phosphorylation was deminished (Fig 4E), consistent with the notion that rapamycin is able to inhibit not only mTORC1 but also mTORC2. Interestingly, *V*. *vulnificus* CFUs were lower in rapamycin treated BMMϕ compared with untreated controls (Fig 4F), which could also contribute to decreased expression of cytokines induced after treatment with rapamycin. Together, these observations suggested that *V*. *vulnificus* contain heat resistant and labile components that can induce multiple cytokine production in macrophages and that mTOR is important for *V*. *vulnificus* proliferation in BMMϕs and may selectively regulate expression of certain but not all cytokines induced by *V*. *vulnificus*.

## Discussion

*V*. *vulnificus* infection causes sepsis in the host and induces inflammation via recruiting and activating immune cells such as neutrophils, monocytes, and macrophages. Extensive work has linked various virulence factors of *V*. *vulnificus* to cell death and modulation of innate immune responses to this pathogen [30, 33–37]. Patients with liver diseases such as cirrhosis are more susceptible to developing *V*. *vulnificus* infection, which can lead to sepsis, sepsis-induced organ failure, and even death [38]. Surprisingly, little is known about the liver phase during *V*. *vulnificus* infection. In this report, we showed that *V*. *vulnificus* CGMCC1.1758 was capable of inducing inflammatory responses in the liver and inflicting liver damage in C57BL/6J mice, manifested by induction of multiple inflammatory cytokines and elevated serum AST and ALT levels. These observations are consistent with a previous report that another *V*. *vulnificus* strain, M06-24/O, is capable of causing liver inflammation and damage in BALB/c mice [31].

As tissue-resident macrophages, Kupffer cells are strategically located to clear bacteria in the liver during infection. Kupffer cells do not obviously proliferate in the steady state [39]. We have found that Kupffer cell numbers drastically increased in mice after *V*. *vulnificus* CGMCC1.1758 infection, which is at least partially caused by proliferation. Like *V*. *vulnificus* infection, systemic *Listeria monocytogenes* infection also results in Kupffer cell proliferation [40]. Thus, Kupffer cell expansion after bacterial infection may represent a means to increase the capacity of tissue-resident macrophages for clearance of invading pathogens. In addition to proliferation, *V*. *vulnificus* infection could increase Kupffer cells via other mechanisms. Our study provides the first evidence that *V*. *vulnificus* can cause mTOR activation. In BMMϕ, either infection with live *V*. *vulnificus* or treatment with heat-inactivated *V*. *vulnificus* or *V*. *vulnificus* lysates induces mTORC1 and mTORC2 activation reflected by 4EBP1 and Akt S473 phosphorylation, respectively. Thus, *V*. *vulnificus* contains component(s) that can induce mTOR activation, although these components and the pattern recognition receptors that lead to mTOR activation remain to be identified. As mentioned earlier, mTOR and its tight regulation play important roles in innate immune responses [11, 15, 16, 20–22]. We have found that *V*. *vulnificus* infection triggered strong inflammatory responses in vivo and *V*. *vulnificus* treatment induced production of multiple cytokines in macrophages. Among these cytokines, GM-CSF, IFNβ, IL-27, and IL-1β production but not IL-6, IL-10, TNF-α, and IL-1α production is dependent on mTOR activity. It would be interesting to further determine the underlying mechanisms by which mTOR controls production of selective cytokines during *V*. *vulnificus* infection.

## Materials and methods

### Ethics statement

This study was carried out in strict accordance with the guidelines of the Zhejiang Provincial Animal Care and Use Administration Office (SYXK-ZJ-2005-0061). We performed all the animal experiments in accordance with protocols approved by the Wenzhou Medical University Animal Care and Use Committee (reference: wydw2014-0009).

### Bacterial strains, mice and cell culture

We purchased C57BL/6J mice from Wenzhou Medical University Laboratory Animal Facility and were maintained under a SPF environment at the Central Animal Laboratory of Wenzhou Medical University. The mice were kept for 5–7 days to acclimatize after transport from the supplier. In the survival experiment, mice were observed every 30 minutes for 24 hours, and mice that survived for 24 hours were euthanized. L-929 cells were purchased from the Cell Bank of the Chinese Academy of Science in Shanghai and cultured them in RPMI1640 containing 10% heat-inactivated fetal bovine serum (Ausvin) and penicillin-streptomycin (50 IU/ml and 50 mg/ml; Beyotime). The China General Microbiological Culture Collection Center provided the *Vibiro vulnificus* CGMCC 1.1758 strain, which we grew at 37°C in brain heart infusion broth (BHI) or on the BHI rabbit blood agar plate.

### In vivo infection

We used i.p. injection to infect female C57BL/6J mice with *V*. *vulnificus*. For survival experiments, we i.p. injected mice with 5×10^7^ or 1×10^8^ CFU of *V*. *vulnificus* suspended in 200 μl PBS or with PBS alone. We euthanized and exsanguinated the mice 6 hours after infection for blood and tissue collection. The liver was fixed it in 4% paraformaldehyde for histological analysis, and froze it in OCT medium (Thermo) for immunofluorescence analysis. To measure *V*. *vulnificus* CFU in the liver, we perfused infected mice with PBS before harvesting their livers. We cultured liver homogenate on BHI blood agar plates at 37°C for 24 hours. We measured serum AST and ALT levels using AST and ALT kits (NjjcBio) following the manufacturer’s protocol.

### Bacterial treatment of BMMϕs in vitro and Western blot

We isolated bone marrow cells and cultured them with 10% L-929 cell culture medium as described previously [15]. We plated BMMϕs in 35 mm dishes and cultured them for 12 hours. We then added live or heat inactivated *V*. *vulnificus* at the indicated multiplicity of infection (MOI) or *V*. *vulnificus* lysates to the cells. We collected supernatants at the indicated times for cytokine quantification. We then subjected the infected cells to lysate preparation and Western blot analysis by following a previous protocol [41]. Anti-phosho-mTOR, anti-phosho-S6K1, anti-phosho-S6, anti-phosho-Akt, anti-phosho-4EBP1, anti-phosho-IKKα/β, anti-phosho-IκBα, and anti-β actin antibodies all came from Cell Signaling Technology.

### Measurement of cytokines by multiplex flow assay

We measured multi-cytokines by using a LEGENDplex multi-analyte flow assay kit (13-plex) for mouse inflammation panel (Biolegend). We assessed the cytokine concentrations in sera, liver homogenates, and supernatants of cultured cells according to the manufacturer’s instructions. We collected data using a BD FACSAria II and analyzed them with LEGENDplex data analysis software (Biolegend).

### BrdU incorporation

We intraperitoneally injected the mice with 1 mg BrdU (BD Bioscience) in 200 μl PBS, then with1×10^8^ CFU of *V*. *vulnificus* two hours later. We harvested the liver from each infected mouse six hours after infection, isolating liver monocytes by following the previous protocol [42]. Briefly, livers were mashed in IMDM with 10% FBS. The upper cell suspension was filtered by nylon mesh and spun at 4°C, 2000 rpm for 5 min. The cell pellet was resuspended in 12 ml 35% Percoll (GE Healthcare), and carefully underlaid with an equal volume of 75% Percoll, and centrifuged at 1000 × *g* for 20 min at room temperature without break. Cells at the interface were collected, washed and resupended in IMDM (10% FBS) for further staining. We stained the cell surface with CD11b and F4/80 (Biolegend) and used a BrdU Flow Kit (BD Bioscience) for BrdU intracellular staining, following the manufacturer’s protocol.

### Flow cytometry

We performed cell surface staining with fluorescence-conjugated antibodies in 2% FBS–PBS. We obtained fluorochrome-conjugated anti-CD11b, anti-F4/80, anti-CD71, and anti-CD98 from Biolegend. We identified cell death using a violet Live/Dead kit (Invitrogen). We performed intracellular staining for Alexa Fluor 647-conjugated p-Akt (Ser473) and p-4EBP1 (Thr37/46) (Cell Signaling Technology) using BD Biosciences Cytofix/Cytoperm and Perm/Wash solutions. We collected data using a BD FACSAria II and analyzed them with FlowJo (Treestar).

### Histological and immunofluorescence analysis

For histology analysis, we imbedded the fixed livers in paraffin, then cut thin sections, and following standard procedures, stained them with hematoxylin and eosin. We quantified the liver histological score of inflammation according to Ishak inflammation score [43, 44]. For immunofluorescence analysis, we cut frozen sections into 8μM slices in a cryostat microtome (Thermo) at -20°C and permeabilized and blocked them. We probed the tissues with appropriate, fluorescently labelled antibodies, including APC-conjugated anti-CD11b, PE-conjugated anti-CD68, and PE-conjugated anti-Ly6G (Biolegend). Finally, we dried the sections and observed them with a Nikon A1 confocal microscope.

### Statistical analysis

We analyzed statistical significance using the Student *t* test and determined survival difference by log-rank survival analysis, performing all statistics using Graphpad Prism 5.0 software. *P* values are defined as follows: *P* < 0.05, *P* < 0.01, and *P* < 0.001.

## References

[1] Blake PA, Merson MH, Weaver RE, Hollis DG, Heublein PC. Disease caused by a marine Vibrio. Clinical characteristics and epidemiology. The New England journal of medicine. 1979;300(1):1–5. doi: 10.1056/NEJM197901043000101 . 758155 10.1056/NEJM197901043000101

[2] Ellington EP, Wood JG, Hill EO. Disease caused by a marine vibrio—Vibrio vulnificus. The New England journal of medicine. 1982;307(26):1642 doi: 10.1056/NEJM198212233072609 . 7144852 10.1056/NEJM198212233072609

[3] Gulig PA, Bourdage KL, Starks AM. Molecular Pathogenesis of Vibrio vulnificus. Journal of microbiology. 2005;43 Spec No:118–31. . 15765065

[4] Kumar H, Kawai T, Akira S. Pathogen recognition by the innate immune system. International reviews of immunology. 2011;30(1):16–34. doi: 10.3109/08830185.2010.529976 . 21235323 10.3109/08830185.2010.529976

[5] Akira S, Uematsu S, Takeuchi O. Pathogen recognition and innate immunity. Cell. 2006;124(4):783–801. doi: 10.1016/j.cell.2006.02.015 . 16497588 10.1016/j.cell.2006.02.015

[6] Bauernfeind FG, Horvath G, Stutz A, Alnemri ES, MacDonald K, Speert D, et al Cutting edge: NF-kappaB activating pattern recognition and cytokine receptors license NLRP3 inflammasome activation by regulating NLRP3 expression. Journal of immunology. 2009;183(2):787–91. doi: 10.4049/jimmunol.0901363 ; PubMed Central PMCID: PMC2824855. 19570822 10.4049/jimmunol.0901363 PMC2824855

[7] Miao EA, Leaf IA, Treuting PM, Mao DP, Dors M, Sarkar A, et al Caspase-1-induced pyroptosis is an innate immune effector mechanism against intracellular bacteria. Nature immunology. 2010;11(12):1136–42. doi: 10.1038/ni.1960 ; PubMed Central PMCID: PMC3058225. 21057511 10.1038/ni.1960 PMC3058225

[8] Netea MG, Nold-Petry CA, Nold MF, Joosten LA, Opitz B, van der Meer JH, et al Differential requirement for the activation of the inflammasome for processing and release of IL-1beta in monocytes and macrophages. Blood. 2009;113(10):2324–35. doi: 10.1182/blood-2008-03-146720 ; PubMed Central PMCID: PMC2652374. 19104081 10.1182/blood-2008-03-146720 PMC2652374

[9] Sharma D, Kanneganti TD. The cell biology of inflammasomes: Mechanisms of inflammasome activation and regulation. The Journal of cell biology. 2016;213(6):617–29. doi: 10.1083/jcb.201602089 ; PubMed Central PMCID: PMC4915194. 27325789 10.1083/jcb.201602089 PMC4915194

[10] Latz E, Xiao TS, Stutz A. Activation and regulation of the inflammasomes. Nature reviews Immunology. 2013;13(6):397–411. doi: 10.1038/nri3452 ; PubMed Central PMCID: PMC3807999. 23702978 10.1038/nri3452 PMC3807999

[11] Weichhart T, Costantino G, Poglitsch M, Rosner M, Zeyda M, Stuhlmeier KM, et al The TSC-mTOR signaling pathway regulates the innate inflammatory response. Immunity. 2008;29(4):565–77. doi: 10.1016/j.immuni.2008.08.012 . 18848473 10.1016/j.immuni.2008.08.012

[12] Byles V, Covarrubias AJ, Ben-Sahra I, Lamming DW, Sabatini DM, Manning BD, et al The TSC-mTOR pathway regulates macrophage polarization. Nature communications. 2013;4:2834 doi: 10.1038/ncomms3834 ; PubMed Central PMCID: PMC3876736. 24280772 10.1038/ncomms3834 PMC3876736

[13] Chapman NM, Chi H. mTOR Links Environmental Signals to T Cell Fate Decisions. Frontiers in immunology. 2014;5:686 doi: 10.3389/fimmu.2014.00686 ; PubMed Central PMCID: PMC4299512. 25653651 10.3389/fimmu.2014.00686 PMC4299512

[14] O'Brien TF, Zhong XP. The role and regulation of mTOR in T-lymphocyte function. Archivum immunologiae et therapiae experimentalis. 2012;60(3):173–81. doi: 10.1007/s00005-012-0171-4 ; PubMed Central PMCID: PMC3376380. 22484804 10.1007/s00005-012-0171-4 PMC3376380

[15] Pan H, O'Brien TF, Zhang P, Zhong XP. The role of tuberous sclerosis complex 1 in regulating innate immunity. Journal of immunology. 2012;188(8):3658–66. doi: 10.4049/jimmunol.1102187 ; PubMed Central PMCID: PMC3324625. 22412198 10.4049/jimmunol.1102187 PMC3324625

[16] Weichhart T, Hengstschlager M, Linke M. Regulation of innate immune cell function by mTOR. Nature reviews Immunology. 2015;15(10):599–614. doi: 10.1038/nri3901 . 26403194 10.1038/nri3901 PMC6095456

[17] Masui K, Tanaka K, Akhavan D, Babic I, Gini B, Matsutani T, et al mTOR complex 2 controls glycolytic metabolism in glioblastoma through FoxO acetylation and upregulation of c-Myc. Cell metabolism. 2013;18(5):726–39. doi: 10.1016/j.cmet.2013.09.013 ; PubMed Central PMCID: PMC3840163. 24140020 10.1016/j.cmet.2013.09.013 PMC3840163

[18] Sarbassov DD, Guertin DA, Ali SM, Sabatini DM. Phosphorylation and regulation of Akt/PKB by the rictor-mTOR complex. Science. 2005;307(5712):1098–101. doi: 10.1126/science.1106148 . 15718470 10.1126/science.1106148

[19] Tato I, Bartrons R, Ventura F, Rosa JL. Amino acids activate mammalian target of rapamycin complex 2 (mTORC2) via PI3K/Akt signaling. The Journal of biological chemistry. 2011;286(8):6128–42. doi: 10.1074/jbc.M110.166991 ; PubMed Central PMCID: PMC3057817. 21131356 10.1074/jbc.M110.166991 PMC3057817

[20] Haidinger M, Poglitsch M, Geyeregger R, Kasturi S, Zeyda M, Zlabinger GJ, et al A versatile role of mammalian target of rapamycin in human dendritic cell function and differentiation. Journal of immunology. 2010;185(7):3919–31. doi: 10.4049/jimmunol.1000296 . 20805416 10.4049/jimmunol.1000296

[21] Zhu L, Yang T, Li L, Sun L, Hou Y, Hu X, et al TSC1 controls macrophage polarization to prevent inflammatory disease. Nat Commun. 2014;5:4696 doi: 10.1038/ncomms5696 . 25175012 10.1038/ncomms5696

[22] Ivanov SS, Roy CR. Pathogen signatures activate a ubiquitination pathway that modulates the function of the metabolic checkpoint kinase mTOR. Nature immunology. 2013;14(12):1219–28. doi: 10.1038/ni.2740 ; PubMed Central PMCID: PMC3839319. 24121838 10.1038/ni.2740 PMC3839319

[23] Jiang Q, Weiss JM, Back T, Chan T, Ortaldo JR, Guichard S, et al mTOR kinase inhibitor AZD8055 enhances the immunotherapeutic activity of an agonist CD40 antibody in cancer treatment. Cancer research. 2011;71(12):4074–84. doi: 10.1158/0008-5472.CAN-10-3968 ; PubMed Central PMCID: PMC3116937. 21540234 10.1158/0008-5472.CAN-10-3968 PMC3116937

[24] Deng W, Yang J, Lin X, Shin J, Gao J, Zhong XP. Essential Role of mTORC1 in Self-Renewal of Murine Alveolar Macrophages. Journal of immunology. 2017;198(1):492–504. doi: 10.4049/jimmunol.1501845 ; PubMed Central PMCID: PMC5173435. 27881705 10.4049/jimmunol.1501845 PMC5173435

[25] Wang Y, Huang G, Zeng H, Yang K, Lamb RF, Chi H. Tuberous sclerosis 1 (Tsc1)-dependent metabolic checkpoint controls development of dendritic cells. Proceedings of the National Academy of Sciences of the United States of America. 2013;110(50):E4894–903. doi: 10.1073/pnas.1308905110 ; PubMed Central PMCID: PMC3864282. 24282297 10.1073/pnas.1308905110 PMC3864282

[26] Sathaliyawala T, O'Gorman WE, Greter M, Bogunovic M, Konjufca V, Hou ZE, et al Mammalian target of rapamycin controls dendritic cell development downstream of Flt3 ligand signaling. Immunity. 2010;33(4):597–606. doi: 10.1016/j.immuni.2010.09.012 ; PubMed Central PMCID: PMC2966531. 20933441 10.1016/j.immuni.2010.09.012 PMC2966531

[27] Pan H, O'Brien TF, Wright G, Yang J, Shin J, Wright KL, et al Critical role of the tumor suppressor tuberous sclerosis complex 1 in dendritic cell activation of CD4 T cells by promoting MHC class II expression via IRF4 and CIITA. Journal of immunology. 2013;191(2):699–707. doi: 10.4049/jimmunol.1201443 ; PubMed Central PMCID: PMC3702379. 23776173 10.4049/jimmunol.1201443 PMC3702379

[28] Jenne CN, Kubes P. Immune surveillance by the liver. Nature immunology. 2013;14(10):996–1006. doi: 10.1038/ni.2691 . 24048121 10.1038/ni.2691

[29] Kashimoto T, Ueno S, Hanajima M, Hayashi H, Akeda Y, Miyoshi S, et al Vibrio vulnificus induces macrophage apoptosis in vitro and in vivo. Infection and immunity. 2003;71(1):533–5. doi: 10.1128/IAI.71.1.533-535.2003 ; PubMed Central PMCID: PMC143416. 12496206 10.1128/IAI.71.1.533-535.2003 PMC143416

[30] Jeong HG, Satchell KJ. Additive function of Vibrio vulnificus MARTX(Vv) and VvhA cytolysins promotes rapid growth and epithelial tissue necrosis during intestinal infection. PLoS pathogens. 2012;8(3):e1002581 doi: 10.1371/journal.ppat.1002581 ; PubMed Central PMCID: PMC3310748. 22457618 10.1371/journal.ppat.1002581 PMC3310748

[31] Liu XF, Wu J, Wang MY, Chen YJ, Cao Y, Hu CJ. Identification of Novel Inflammatory Cytokines and Contribution of Keratinocyte-Derived Chemokine to Inflammation in Response to Vibrio vulnificus Infection in Mice. Inflammation. 2015;38(5):1864–73. doi: 10.1007/s10753-015-0166-5 . 25862020 10.1007/s10753-015-0166-5

[32] Ali S, Mann DA. Signal transduction via the NF-kappaB pathway: a targeted treatment modality for infection, inflammation and repair. Cell biochemistry and function. 2004;22(2):67–79. doi: 10.1002/cbf.1082 . 15027095 10.1002/cbf.1082

[33] Kim K, Kim NJ, Kim SY, Kim IH, Kim KS, Lee GR. Cyclo(Phe-Pro) produced by the human pathogen Vibrio vulnificus inhibits host innate immune responses through the NF-kappaB pathway. Infection and immunity. 2015;83(3):1150–61. doi: 10.1128/IAI.02878-14 ; PubMed Central PMCID: PMC4333476. 25561711 10.1128/IAI.02878-14 PMC4333476

[34] Lee BC, Kim MS, Choi SH, Kim TS. Involvement of capsular polysaccharide via a TLR2/NF-kappaB pathway in Vibrio vulnificus-induced IL-8 secretion of human intestinal epithelial cells. International journal of molecular medicine. 2010;25(4):581–91. . 20198307 10.3892/ijmm_00000380

[35] Lee SE, Kim SY, Jeong BC, Kim YR, Bae SJ, Ahn OS, et al A bacterial flagellin, Vibrio vulnificus FlaB, has a strong mucosal adjuvant activity to induce protective immunity. Infection and immunity. 2006;74(1):694–702. doi: 10.1128/IAI.74.1.694-702.2006 ; PubMed Central PMCID: PMC1346682. 16369026 10.1128/IAI.74.1.694-702.2006 PMC1346682

[36] Lee SJ, Jung YH, Oh SY, Song EJ, Choi SH, Han HJ. Vibrio vulnificus VvhA induces NF-kappaB-dependent mitochondrial cell death via lipid raft-mediated ROS production in intestinal epithelial cells. Cell death & disease. 2015;6:1655 doi: 10.1038/cddis.2015.19 ; PubMed Central PMCID: PMC4669806. 25695598 10.1038/cddis.2015.19 PMC4669806

[37] Lee SJ, Jung YH, Song EJ, Jang KK, Choi SH, Han HJ. Vibrio vulnificus VvpE Stimulates IL-1beta Production by the Hypomethylation of the IL-1beta Promoter and NF-kappaB Activation via Lipid Raft-Dependent ANXA2 Recruitment and Reactive Oxygen Species Signaling in Intestinal Epithelial Cells. Journal of immunology. 2015;195(5):2282–93. doi: 10.4049/jimmunol.1500951 . 26223656 10.4049/jimmunol.1500951

[38] Nazir S, Brown K, Shin AK, Donato AA. Vibrio vulnificus infection and liver cirrhosis: a potentially lethal combination. BMJ case reports. 2016;2016 doi: 10.1136/bcr-2016-214772 . 27151052 10.1136/bcr-2016-214772 PMC4885366

[39] Gosselin D, Link VM, Romanoski CE, Fonseca GJ, Eichenfield DZ, Spann NJ, et al Environment drives selection and function of enhancers controlling tissue-specific macrophage identities. Cell. 2014;159(6):1327–40. doi: 10.1016/j.cell.2014.11.023 ; PubMed Central PMCID: PMC4364385. 25480297 10.1016/j.cell.2014.11.023 PMC4364385

[40] Bleriot C, Dupuis T, Jouvion G, Eberl G, Disson O, Lecuit M. Liver-resident macrophage necroptosis orchestrates type 1 microbicidal inflammation and type-2-mediated tissue repair during bacterial infection. Immunity. 2015;42(1):145–58. doi: 10.1016/j.immuni.2014.12.020 . 25577440 10.1016/j.immuni.2014.12.020

[41] Gorentla BK, Wan CK, Zhong XP. Negative regulation of mTOR activation by diacylglycerol kinases. Blood. 2011;117(15):4022–31. doi: 10.1182/blood-2010-08-300731 ; PubMed Central PMCID: PMC3087529. 21310925 10.1182/blood-2010-08-300731 PMC3087529

[42] Wu J, Shin J, Xie D, Wang H, Gao J, Zhong XP. Tuberous sclerosis 1 promotes invariant NKT cell anergy and inhibits invariant NKT cell-mediated antitumor immunity. Journal of immunology. 2014;192(6):2643–50. doi: 10.4049/jimmunol.1302076 ; PubMed Central PMCID: PMC3965184. 24532578 10.4049/jimmunol.1302076 PMC3965184

[43] Farrell GC, Chitturi S, Lau GK, Sollano JD, Asia-Pacific Working Party on N. Guidelines for the assessment and management of non-alcoholic fatty liver disease in the Asia-Pacific region: executive summary. Journal of gastroenterology and hepatology. 2007;22(6):775–7. doi: 10.1111/j.1440-1746.2007.05002.x . 17565629 10.1111/j.1440-1746.2007.05002.x

[44] Ishak K, Baptista A, Bianchi L, Callea F, De Groote J, Gudat F, et al Histological grading and staging of chronic hepatitis. Journal of hepatology. 1995;22(6):696–9. . 7560864 10.1016/0168-8278(95)80226-6

